# Identification of New Tumor-Related Gene Mutations in Chinese Gastrointestinal Stromal Tumors

**DOI:** 10.3389/fcell.2021.764275

**Published:** 2021-11-03

**Authors:** Yuyang Feng, Surui Yao, Zhening Pu, Han Cheng, Bojian Fei, Jian Zou, Zhaohui Huang

**Affiliations:** ^1^ Wuxi Cancer Institute, Affiliated Hospital of Jiangnan University, Wuxi, China; ^2^ Laboratory of Cancer Epigenetics, Wuxi School of Medicine, Jiangnan University, Wuxi, China; ^3^ Center of Clinical Research, The Affiliated Wuxi People’s Hospital of Nanjing Medical University, Wuxi, China; ^4^ Department of Gastrointestinal Surgery, Affiliated Hospital of Jiangnan University, Wuxi, China

**Keywords:** gastrointestinal stromal tumors, mutation, whole exome sequencing, KIT, PDGFRA, metabolism, DNA repair

## Abstract

Gastrointestinal stromal tumors (GISTs) are the most common mesenchymal tumors of the gastrointestinal tract. As the main GIST drivers, gain-of-function mutations in *KIT* or *PDGFRA* are closely associated with not only tumor development and progression but also therapeutic response. In addition to the status of KIT and PDGFRA, little is known about other potential GIST-related genes. In this study, we identified the mutation profiles in 49 KIT-mutated GIST tumors using the whole exome sequencing (WES) method. Furthermore, some representative mutations were further validated in an independent GIST cohort using the SNaPshot SNP assay. We identified extensive and diverse mutations of KIT in GIST, including many undescribed variants. In addition, we revealed some new tumor-related gene mutations with unknown pathogenicity. By enrichment analyses of gene function and protein-protein interaction network construction, we showed that these genes were enriched in several important cancer- or metabolism-related signaling pathways, including PI3K-AKT,RTK-RAS, Notch, Wnt, Hippo, mTOR, AMPK, and insulin signaling. In particular, DNA repair-related genes, including *MLH1*, *MSH6*, *BRCA1*, *BRCA2*, and *POLE*, are frequently mutated in GISTs, suggesting that immune checkpoint blockade may have promising clinical applications for these GIST subpopulations. In conclusion, in addition to extensive and diverse mutations of *KIT*, some genes related to DNA-repair and cell metabolism may play important roles in the development, progression and therapeutic response of GIST.

## Introduction

Gastrointestinal stromal tumors (GIST) are the most common mesenchymal tumors of the digestive tract, and are mainly driven by activating mutations in KIT (also known as CD117) or platelet-derived growth factor A (PDGFRA), accounting for 0.1–3% of all gastrointestinal tumors. The status of KIT or PDGFRA is closely associated with GIST development, progression and therapeutic response. GISTs harboring the same *KIT/PDGFRA* mutations often display different malignant features and response to therapy, suggesting that there are other potential GIST-related genes that influence the biological phenotypes or prognosis of GIST patients (Li and Raut, 2019); however, little is known about the mutation profiles at the genome level in GIST.

Imatinib, a selective small molecule inhibitor for tyrosine kinases, is the only first-line drug for GIST. The oncogenic tyrosine kinase activity of *KIT* and *PDGFRA* was significantly inhibited by imatinib, resulting in obviously improved prognosis of GIST patients harboring oncogenic mutations in *KIT/PDGFRA*. The efficacy of imatinib depends on the genotypes of *KIT* or *PDGFRA*. However, the drug response to some *KIT/PDGFRA*-mutant GISTs is poor due to primary or secondary resistance. Mutation analyses of *KIT* and *PDGFRA* genes, especially the exon 11/9 of *KIT* and exon 18 of *PDGFRA*, were usually performed using PCR amplification and Sanger sequencing assays. Although some mutations in *KIT/PDGFRA* have been suggested to be associated with the sensitivity of GIST to imatinib therapy, obvious differences in drug response were observed in some patients even with “the same” mutation in *KIT/PDGFRA*. We speculated that some unknown mutations in *KIT/PDGFRA* or other genes, which failed to be detected by traditional PCR-based Sanger sequencing, may also take part in the development and therapeutic response of GIST.

In this study, we performed an omics-based analysis in 47 Chinese GIST patients treated with imatinib using whole exome sequencing (WES). Our aims were to comprehensively analyze genomic changes in Chinese GISTs and to identify rare novel gene mutations in *KIT/PDGFRA* or other tumor-related genes that may take part in the development and chemoresistance of GIST from an omics viewpoint.

## Materials and Methods

### Clinical Samples

A total of 49 formalin-fixed, paraffin-embedded GIST tissues were collected from 47 patients receiving imatinib therapy at Affiliated Hospital of Jiangnan University, and were subjected to WES analyses. Of these 49 tumors, 47 were collected before imatinib therapy, and two metastatic tumors were collected from patients who had received adjuvant imatinib treatment. In addition, an independent cohort of 97 GIST patients was enrolled for the validation of WES results. The study protocols were approved by the Clinical Research Ethics Committees of Affiliated Hospital of Jiangnan University (No: LS2014065). The detailed clinical information of the two GIST cohorts is listed in Table 1 and Supplementary Table S1.

**TABLE 1:** Clinical pathological features of 49 GISTs.

case No	Gender	Age	Tumor site	Tumor size (cm)	Mitotic count/(50HPF)	Risk classification	Primary/metastasis
S01	M	59	Small intestine	12	>5	High	Metastasis
S02	M	45	Small intestine	10	<5	High	Primary
S04	M	44	Rectal	6	>5	High	Primary
S05	M	46	Stomach	10	>10	High	Primary
S07	M	70	Stomach	10	>5	High	Primary
S08	M	59	Stomach	7	>5	High	Primary
S09	M	59	Stomach	7	>5	High	Primary
S10	M	60	Stomach	8	>5	High	Primary
S11	F	62	Small intestine	9	>5	High	Primary
S13	M	75	Small intestine	2	>5	High	Metastasis
S14	M	51	Abdominal cavity	15	<5	High	Primary
S15	M	53	Abdominal cavity	21	>10	High	Metastasis
S17	F	38	Stomach	11	<10	High	Primary
S18	F	41	Abdominal cavity	30	>5	High	Metastasis
S25	M	74	Small intestine	10	>10	High	Primary
S26	F	38	Small intestine	8	<5	High	Primary
S29	F	61	Small intestine	6	<5	High	Primary
S3	F	55	Stomach	5	>5	Intermediate	Primary
S30	F	38	Abdominal cavity	15	<10	High	Metastasis
S31	M	60	Stomach	15	<10	High	Primary
S32	M	66	Small intestine	14	>10	High	Primary
S35	M	51	Small intestine	10	<5	High	Primary
S36	F	58	Small intestine	5	<5	Low	Primary
S37	F	64	Small intestine	12	<5	High	Primary
S38	F	59	Stomach	10	<5	Intermediate	Primary
S39	M	54	Stomach	9	<5	Intermediate	Primary
S41	M	80	Small intestine	5	>5	High	Primary
S42	M	54	Small intestine	9	<5	High	Primary
S43	M	74	Stomach	9	>5	High	Primary
S44	M	72	Small intestine	17	<5	High	Primary
S46	M	42	Stomach	18	<5	High	Primary
S47	F	53	Small intestine	9	>5	High	Primary
S49	F	59	Stomach	7	>5	High	Primary
S51	F	61	Small intestine	10	<5	High	Primary
S52	M	44	Abdominal cavity	14	<5	High	Primary
S53	M	56	Stomach	8	<5	Intermediate	Primary
S55	F	64	Small intestine	12	>10	High	Primary
S57	F	59	Stomach	5	<10	Intermediate	Primary
S58	F	77	Small intestine	5	<5	Low	Primary
S59	F	71	Stomach	6	>5	Intermediate	Primary
S60	F	49	Stomach	5	<5	Intermediate	Primary
S61	M	73	Stomach	13	<5	High	Primary
S65	F	56	Stomach	9	<5	Intermediate	Primary
S66	M	46	Small intestine	3	<5	Low	Primary
S67	M	47	Stomach	9	<5	Intermediate	Primary
S68	M	49	Rectal	5	<5	Low	Primary
S72	M	33	Small intestine	4	<5	Low	Primary
S75	M	59	Abdominal cavity	-	<5	High	Metastasis
S77	M	70	Small intestine	8	<10	High	Metastasis

### WES and Bioinformatics Analyses

Genomic DNA was purified from formalin-fixed, paraffin-embedded (FFPE) GIST tissues using QIAamp DNA FFPE Tissue Kit (QIAGEN, Germany), and DNA quality was evaluated using a NanoDrop 2000 spectrophotometer (Thermo Fisher, United States). A SureSelectXT Human All Exon V6 (Agilent Technologies, United States) was used for exome-sequencing library preparation according to the manufacturer’s instructions. DNA sequencing was performed on the HiSeq 2,500 system (Illumina, United States) at the Shanghai Biotechnology Corporation (SHBIO, China). Raw sequencing reads were filtered to trim adapters and low quality reads using Trimmomatic-0.3.2 under PE module. All the qualified readswere processed with an in-house bioinformatics pipeline, which followed the best practicesteps suggested by Genome Analysis Toolkit (GATK) (DePristo et al., 2011). Briefly, the clean sequence reads were aligned to the human Hg19 reference genome using Burrows–Wheeler Aligner (BWA-MEM v0.7.12). PCR duplicates were removed by Picard v1.141. Afterinitial quality control, all eligible sequences were determined for regional realignment andbase quality recalibration with GATK v3.5. Variants, including single nucleotidevariants (SNV), insertions and deletions, were then called using HaplotypeCaller module of GATK v3.5. All variants were functionally annotated by ANNOVAR (http://annovar.openbioinformatics.org/en/latest/) using the 1,000 Genomes Project (http://www.1000genomes.org/), Exome Aggregation Consortium (ExAC) Browser, ClinVar, and MutationTaster (http://www.mutationtaster.org/). Finally, all remaining mutations were manually checked using the Integrated Genome Viewer (IGV) (http://www.broadinstitute.org/igv). The WES data had been uploaded to Science Data Bank (ScienceDB) (http://www.doi.org/0.11922/sciencedb.01155).

### Mutation Analyses of Cancer-Associated Genes

Oncogenic signaling pathways and mutation analyses mainly focused on 368 tumor-related genes, including genes from MSK-IMPACT™ (https://www.mskcc.org/msk-impact) (Cheng et al., 2015) (Supplementary Table S2) and those reported on GIST (Heinrich et al., 2006; Belinsky et al., 2015; Campanella et al., 2015; Hechtman et al., 2015; Klinke et al., 2015; Huang et al., 2016; Zehir et al., 2017). Additional selection strategies for potential functional non-somatic alterations include: 1) they were nonsynonymous SNVs; 2) exonic and splice-site variants; 3) variants not located in the segmental duplication region marked by UCSC browser (http://genome.ucsc.edu/); 4) their AF (allele frequency) < 0.05 in the 1,000 genomes project or ExAC; 5) variants predicted to be disease_causing or conservative by MutationTaster, SIFT (http://sift.jcvi.org) score and PolyPhen-2 score (http://genetics.bwh.harvard. edu/pph2/); and 6) variants not included in the Noflag SNP138 database. The lollipop plots and oncoprints were generated using cBioPortal online tools (https://www.cbioportal.org/).

### Copy Number Variant (CNV) and Tumor Mutation Burden (TMB) Analyses

The exome hidden Markov model (XHMM) tool was used for CNV detection in the WES sequencing data. Tumor mutational burden (TMB) was calculated as previously described (Melendez et al., 2018).

### Mutation Signature Analyses

Somatic Signatures Package (R Version 4.1.0) was used to analyze mutation signature. We performed mutation analyses using somatic mutation from 49 GIST samples annotated by Cosmic70 database. For analyses of mutation signatures, mutations were classified into six types determined by the six possible substitutions (A:T > C:G, A:T > G:C, A:T > T:A, C:G > A:T, C:G > G:C, and C:G > T:A) and the 16 combinations of flanking (5′and 3′) nucleotides. Single base substitutions (SBS) signature analyses mainly included COSMIC Signatures 1 to 30 (https://cancer.sanger.ac.uk/signatures/sbs/).

### Sanger DNA Sequencing

Some representative or novel variants revealed in GISTs by WES analyses were further validated using PCR-based Sanger DNA sequencing. The related sequences of PCR primers are listed in Supplementary Table S3. PCR amplifications were performed using Takara Ex Taq Hot Start polymerase (Takara, Japan) according to the manufacturer’s instructions. PCR products were subjected to Sanger DNA sequencing at the Shanghai Biotechnology Corporation (SHBIO, China).

### Protein-Protein Interaction and Pathway Enrichment Analyses

PPI networks were constructed using STRING software (https://string-db.org/). Target genes were subjected to pathway enrichment analyses of gene function using Kyoto Encyclopedia of Genes and Genomes (KEGG) and Gene Ontology (GO) pathway databases as we previously described (Zhang et al., 2019), and enriched pathways with *p* < 0.05 were selected for subsequent analyses. In addition, the pathway enrichment of mutant genes in ten canonical oncogenic signaling pathways was also conducted using Package ‘maftools’ Version 2.4.12 of R software (Sanchez-Vega et al., 2018).

### SNaPshot SNP Assay

To validate the WES results, selected SNVs were analyzed in an expanded GIST cohort using a multiplex SNaPshot SNP assay as previously described (Wang et al., 2012). In brief, genomic DNA was purified from 97 formalin-fixed, paraffin-embedded GIST tissues. These DNA samples were used for multiplex PCR in 20 µl reaction mixtures. The PCR procedure was as follows: an initial melting step of 120 s at 95°C; 11 cycles of 20 s at 94°C, 40 s at 65°C and 90 s at 72°C; 24 cycles of 20 s at 94°C, 30 s at 59°C and 90 s at 72°C; and a final elongation step of 120 s at 72°C. After treatment with 5 U SAP and 2 U exonuclease I, these PCR products were subjected to multiplex single-base extension reactions using ABI PRISM SNaPshot Multiplex Kit (ABI, United States). The program of extension reactions was 60 s at 96°C; 28 cycles of 10 s at 96°C, 5 s at 50°C, and 30 s at 60°C. Finally, the extension products were treated with SAP and analyzed using Applied Biosystems™ 3730xl DNA Analyzer (ABI). The primer sequences used for SNaPshot SNP assay are listed in Supplementary Table S4.

## Results

### WES Analyses of DNA Samples From FFPE GIST Tissues

**FIGURE 1:**
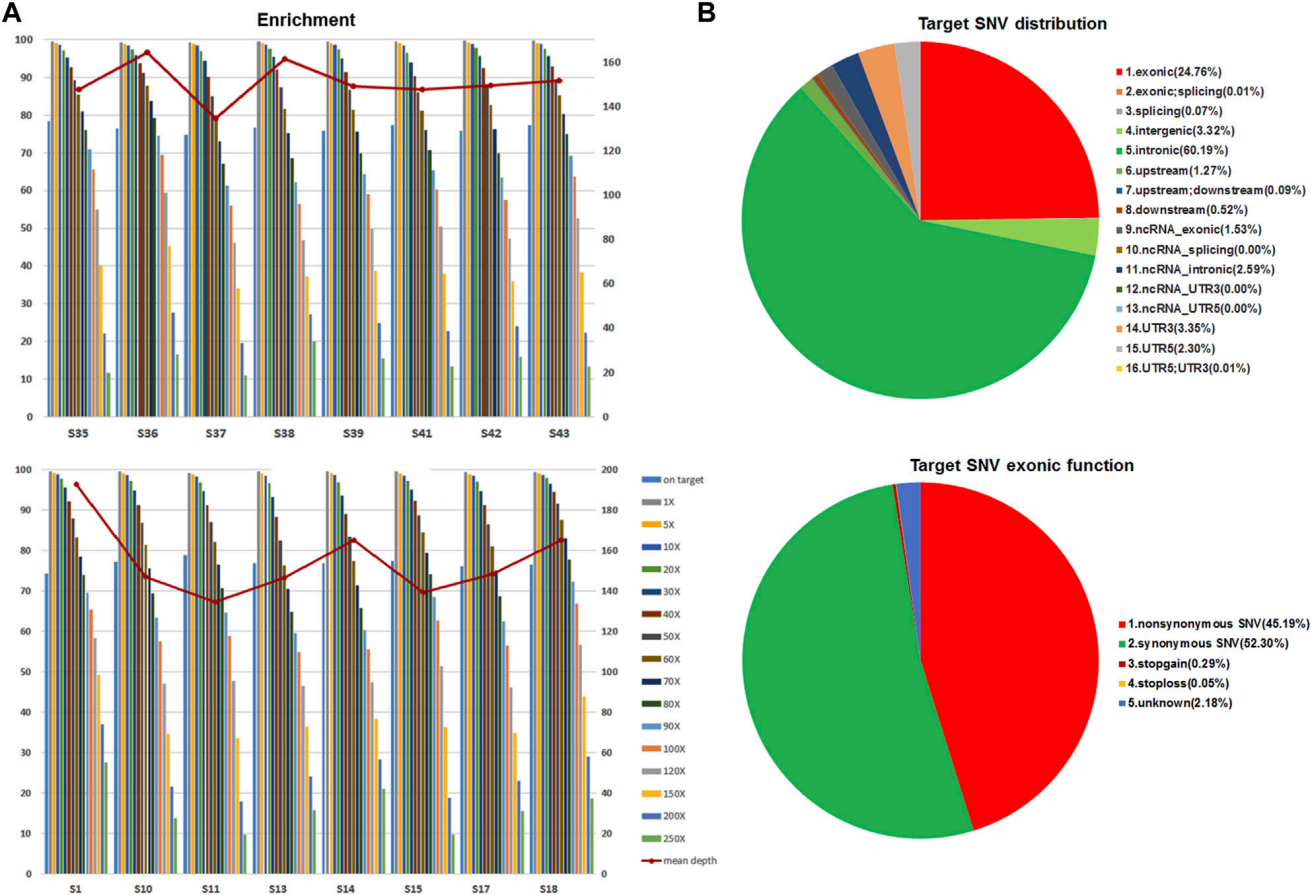
Mean enrichment and depth of WES on FFPE GIST tissues and SNP distribution. (**A**) Representative results of unique mapped ratios and length of WES assay on 49 FFPE GIST tissues. (**B**) The mean target SNV distribution in 49 GIST tumors.

Forty-nine GIST tissues, including 42 primary tumors and 7 metastatic tumors, were retrospectively collected from 47 Chinese GIST patients for WES analyses. Of these tumors, 20 originated from stomach, 21 had an intestinal origin, two were from the rectum, and 6 were taken from the abdominal cavity (Table 1). WES technology was applied to identify variants in 232,406 target regions in the human genomes of these tumors. After filtering steps, the median total reads (good read) was 114.09 million, and the median total and unique mapped ratios were 99.63 and 85.43%, respectively. The median value of the mean depth in the target region was 152×, and the median value for the percent of paired reads on target region was 76.55% (Figure 1A and Supplementary Table S5). The overall coverage statistics for each case are shown in Supplementary Table S5. In addition, the mean target SNV distribution of these 49 samples is shown in Figure 1B. Together, these data suggest that we could obtain solid WES results using DNA samples of FFPE tumor tissues.

### Extensive and Diverse Mutations of KIT in GIST

**FIGURE 2:**
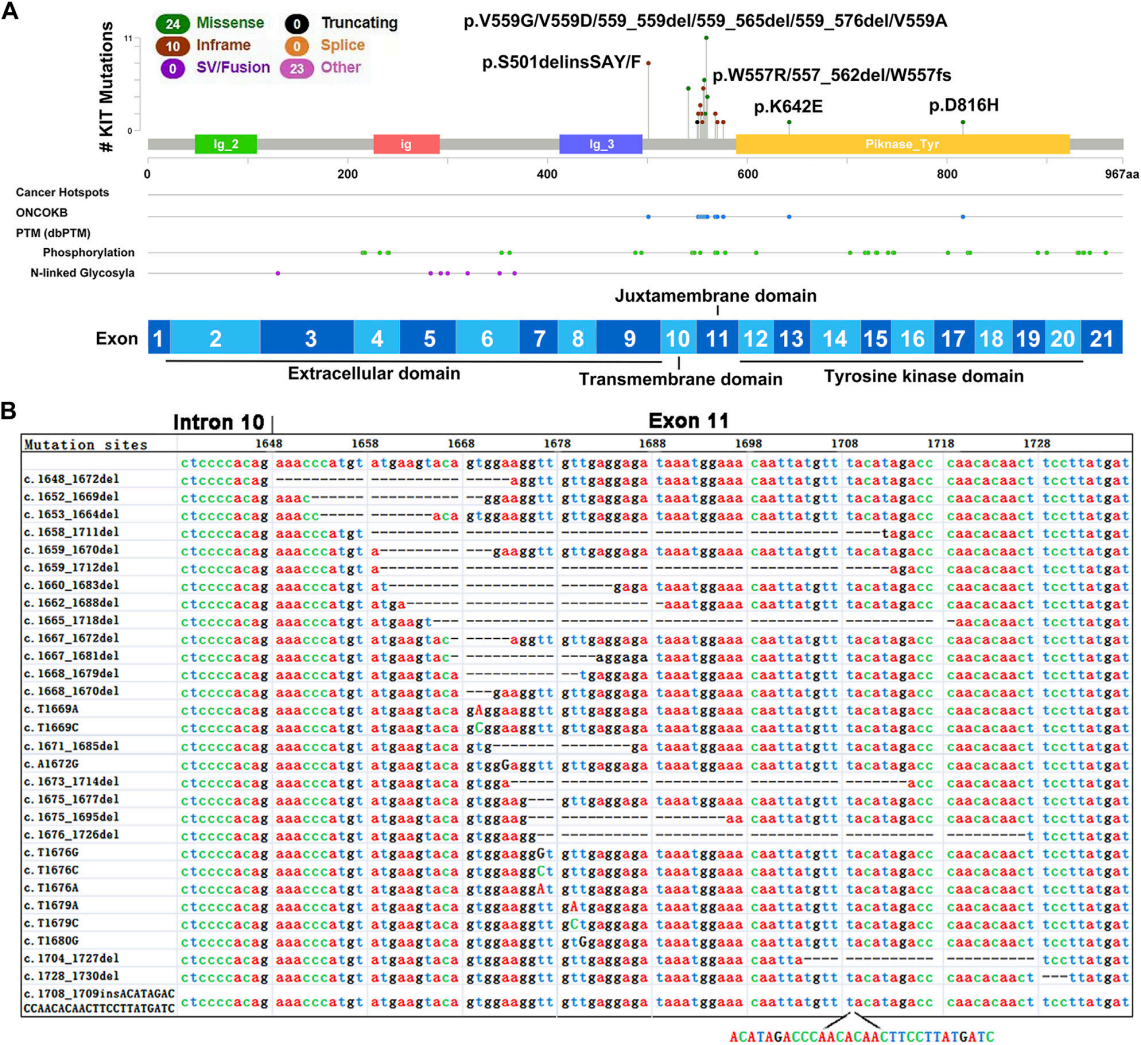
Mutations identified in exon 11 of *KIT* in GIST tumors. **(A)** The lollipop of mutations in *KIT*. **(B)** Mutations identified in exon 11 of *KIT* in 49 GIST tumors.

Previous analyses using PCR-based Sanger DNA sequencing showed that these 49 tumors harbored mutations in exons 9 (9/49), 10 (6/49), 11 (38/49), 13 (1/49) or 17 (1/49) of *KIT* (Figure 2A). Our WES results confirmed these mutations and revealed extensive and diverse mutations in different exons of *KIT*, including nucleotide deletions in 21 cases (42.86%), missense mutations in 24 cases (48.98%) and insertions in 12 cases (24.49%) (Table 2).

**TABLE 2:** *KIT* mutations in 49 GISTs.

Exon	Mutation site	Amino acid change	Mutation types	Mutation ratio% (n/n)	
9	c.1502_1503insTGCCTT	p.S501delinsSAF	nonframeshift insertion	2.04% (1)	
9	c.1502_1503insTGCCTA	p.S501delinsSAY	nonframeshift insertion	16.32% (8)	
10	c.T1679A	p.A560N	missense	2.04% (1)	
10	c.A1621C	p.M541L	missense	10.20% (5)	
11	c.1648_1672del	p.K550fs	frameshift deletion	2.04% (1)	
11	c.1652_1669del	p.551_557del	nonframeshift deletion	2.04% (1)	
11	c.1653_1664del	p.551_555del	nonframeshift deletion	2.04% (1)	
11	c.1658_1711del	p.553_571del	nonframeshift deletion	2.04% (1)	
11	c.1659_1670del	p.553fs	frameshift deletion	2.04% (1)	
11	c.1659_1712del	p.553_572del	nonframeshift deletion	2.04% (1)	
11	c.1660_1683del	p.554_561del	nonframeshift deletion	2.04% (1)	
11	c.1662_1688del	p.554_563del	nonframeshift deletion	2.04% (1)	
11	c.1665_1718del	p.555_573del	nonframeshift deletion	2.04% (1)	
11	c.1667_1672del	p.556_558del	nonframeshift deletion	4.08% (2)	
11	c.1667_1681del	p.556_561del	nonframeshift deletion	4.08% (2)	
11	c.1668_1679del	p.556_560del	nonframeshift deletion	2.04% (1)	
11	c.1668_1670del	p.W557fs	frameshift deletion	2.04% (1)	
11	c.T1669A	p.W557R	missense	6.12% (3)	
11	c.T1669C	p.W557R	missense	2.04% (1)	
11	c.1671_1685del	p.557_562del	nonframeshift deletion	2.04% (1)	
11	c.A1672G	p.K558E	missense	2.04% (1)	
11	c.1673_1714del	p.558_572del	nonframeshift deletion	2.04% (1)	
11	c.1675_1677del	p.559_559del	nonframeshift deletion	2.04% (1)	
11	c.1675_1695del	p.559_565del	nonframeshift deletion	2.04% (1)	
11	c.1676_1726del	p.559_576del	nonframeshift deletion	2.04% (1)	
11	c.T1676G	p.V559G	missense	4.08% (2)	
11	c.T1676C	p.V559A	missense	6.12% (3)	
11	c.T1676A	p.V559D	missense	6.12% (3)	
11	c.T1679A	p.V560D	missense	2.04% (1)	
11	c.T1679C	p.V560E	missense	2.04% (1)	
11	c.T1680G	p.V560E	missense	2.04% (1)	
11	c.1704_1727del	p.568_576del	nonframeshift deletion	4.08% (2)	
11	c.1708_1709insACATAGACCCAACACAACTTCCTTATGATC	p.Y570delinsYIDPTQLPYDH	nonframeshift insertion	2.04% (1)	
11	c.1728_1730del	p.576_577del	nonframeshift deletion	2.04% (1)	
13	c.A1924G	p.K642E	missense	2.04% (1)	
17	c.G2446C	p.D816H	missense	2.04% (1)	

As expected, frequent and various mutations were identified in *KIT* exon 11, which encodes the regulatory juxtamembrane domain of the enzyme (Figure 2A), and most of them were unreported (Table 2 and Figure 2B). A total of 30 types of mutations of exon 11, included nucleotide deletions between codons 551 and 577 in 23 tumors (46.94%), missense mutations in 16 tumors (32.65%) and insertions in 1 case (2.04%). Interestingly, one tumor harbored two different mutations in exon 11 (c.1668_1670del and c.1672A > G). In contrast, the mutations in *KIT* exon 9 were highly homogeneous, and 9 tumors harbored two insertion alterations at the same site (8 tumors with 1502_1503insTGCCTA, and one with 1502_1503insTGCCTT), resulting in the same insert of two amino acids (Cys-Leu). Several SNVs in exon 10 were also observed, including five tumors harboring a missense mutation (c.A1621C, p.M541L) and one tumor carrying one potential pathogenic mutation (c.T1679A, p.A560N). Interestingly, all six tumors harbored *KIT* exon 11 mutations simultaneously.

**FIGURE 3:**
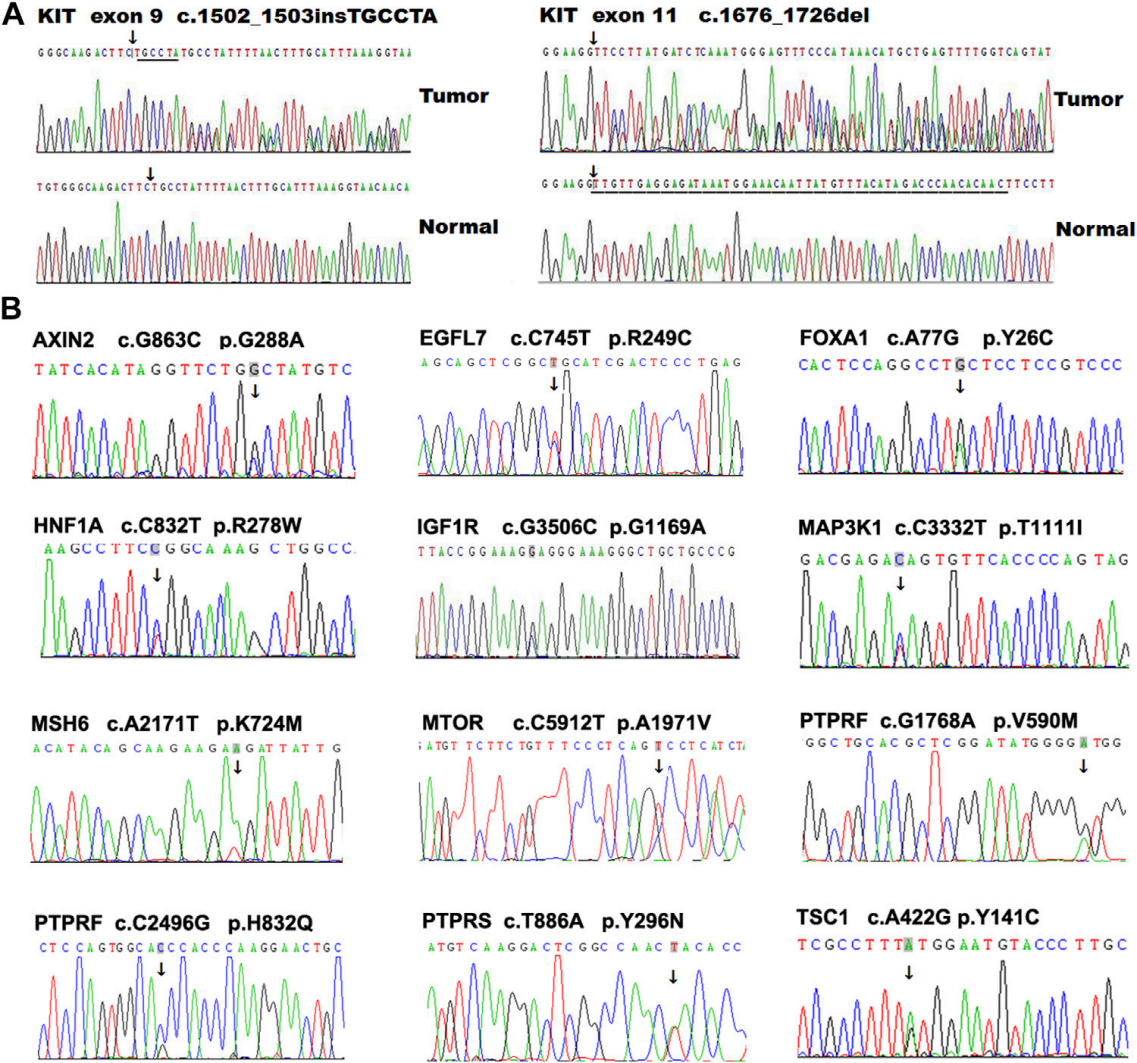
Mutation validation in GIST tumors using PCR-based Sanger DNA sequencing. Some representative variations located in *KIT.* **(A)** and other genes **(B)** were validated using PCR-based Sanger DNA sequencing in GIST tumors.

GISTs carrying mutations in *KIT* exons 13 and 17 are reported resistant to imatinib (Heinrich et al., 2006). We observed that a metastatic GIST tumor harbored two different *KIT* mutations, including a deletion mutation in exon 11 (c.1675_1695del) and a missense mutation in exon 17 (c.G2446C, p. D816H), suggesting that acquired mutation in exon 17 occurred in the metastatic tumor compared with the primary tumor. One exon 11-mutant (c.T1676C, p.V559A) tumor also carried a novel synonymous SNV(c.C2235T, p.G745G) in exon 16. Some representative *KIT* mutations were further validated using Sanger sequencing (Figure 3A). In addition, a functional unknown SNV (rs2291591), located in the exon 17 of *KIT* (NM_001347827, c. C2345T, p.T782M), was also observed in 19.1% (9/47) of GIST patients.

It is rare that a GIST tumor harbors mutations in both *KIT* and *PDGFRA,* simultaneously; however, one GIST case (S09) carries two GIST driver mutations located in exon 11 of *KIT* (c.T1676C, p.V559A) and in exon 18 of *PDGFRA* (c.A2525T, p.D842V).

### Mutation Screening for Potentially GIST-Related Genes

In addition to *KIT* and *PDGFRA*, several genes, including *SDHA*, *AURKA*, *RAS*, *NF1* and *BRAF*, have also been reported to take part in the development of GIST (von Mehren and Joensuu, 2018) (Supplementary Figure S1). To further investigate other genes potentially regulating the development and progression of GIST, we first analyzed the SNV profiles in 368 tumor-related genes in our GIST cohort, and revealed 31 missense mutations in 28 genes (Table 3). Interestingly, in this *KIT*-mutant GIST cohort, several mutations were also observed in some GIST-related genes that were previously identified in *KIT/PDGFRA*-negative GIST. For example, nine tumors (18.37%, 9/49) harbored a potential risk SNP (rs2273535, c. T91A, p. F31I) in *AURKA*, which is associated with an increased risk of digestive tract cancers (Hienonen et al., 2006; Ju et al., 2006) and with early adverse reactions of the gastrointestinal tract in cervical cancer patients treated with radiation therapy (Ishikawa et al., 2011). Two alterations in *IGF1R*, including a novel SNV (c.G3506C, p. G1169A) and a known SNV (rs45526336, c.G3847A, p.E1283K), were identified in one patient who harbored a *KIT* exon 11 mutation (c.1658-1711del) (Figure 3B). In addition, a novel missense mutation (c.C6799G, p.Q2267E) located in exon 45 of *NF1* and a known SNP (*NF1* c.8515G > A, p.V2839M) was observed in one case.

**TABLE 3:** Potential tumor-related mutations identified in GIST.

Gene	Chrom	Position	Transcript	Mutation site	Amino acid change	Mutation type	CADD phred	MutationTaster score	MutationTaster pred	Polyphen2score	SIFTscore	Mutation ratio n (%)	Notes^a^
AXIN2	chr17	63545731	NM_004655.3	c.G863C	p.G288A	missense	23.70	1.00	Disease_causing	1.00	0.01	1 (2.04%)	
BRCA1	chr17	41244982	NM_007297	c.T2425C	p.Y809	missense	16.53	1.00	Disease_causing	0.96	0.01	3 (6.12%)	
CDK12	chr17	37618577	NM_016507	c.G253T	p.D85Y	missense	20.20	1.00	Disease_causing	1.00	0.01	1 (2.04%)	
EGFL7	chr9	139566486	NM_201446	c.C745T	p.R249C	missense	20.90	1.00	Disease_causing	1.00	0.00	1 (2.04%)	
FOXA1	chr14	38061912	NM_004496	c.A77G	p.Y26C	missense	20.60	1.00	Disease_causing	1.00	0.00	1 (2.04%)	
IGF1R	chr15	99486200	NM_000875	c.G3506C	p.G1169A	missense	35.00	1.00	Disease_causing	0.93	0.00	2 (4.08%)	
INSR	chr19	7125488	NM_000208	c.A3028G	p.K1010E	missense	28.90	1.00	Disease_causing	0.88	0.00	1 (2.04%)	
IRS1	chr2	227662095	NM_005544	c.C1360T	p.P454S	missense	21.30	1.00	Disease_causing	1.00	0.01	1 (2.04%)	
IRS2	chr13	110435733	NM_003749	c.C2668G	p.P890A	missense	15.49	1.00	Disease_causing	0.88	0.02	1 (2.04%)	
MLH1	chr3	37053562	NM_001167619	c.C649T	p.R217C	missense	25.10	1.00	Disease_causing	1.00	0.00	1 (2.04%)	
MLH1	chr3	37067240	NM_000249	c.T428A	p.V143D	missense	31.00	1.00	Disease_causing	1.00	0.00	6 (12.24%)	
PAX5	chr9	37020795	NM_016734	c.A50G	p.H17R	missense	23.70	1.00	Disease_causing	1.00	0.00	1 (2.04%)	
PTPRS	chr19	5245850	NM_130853	c.T886A	p.Y296N	missense	23.70	1.00	Disease_causing	1.00	0.00	2 (4.08%)	
RFWD2	chr1	175958527	NM_022457	c.G1098C	p.K366N	missense	19.36	1.00	Disease_causing	0.99	0.02	1 (2.04%)	
SOX17	chr8	55370975	NM_022454	c.C277G	p.L93V	missense	18.37	1.00	Disease_causing	1.00	0.00	1 (2.04%)	
ARID1B	chr6	157099680	NM_017519	c.G617A	p.G206D	missense	12.61	0.62	Disease_causing	0.98	0.00	1 (2.04%)	
DNMT1	chr19	10249155	NM_001379	c.G4027A	p.V1343M	missense	28.60	1.00	Disease_causing	1.00	0.03	1 (2.04%)	
EPHB1	chr3	134851573	NM_004441	c.C979T	p.R327C	missense	16.93	1.00	Disease_causing	1.00	0.00	1 (2.04%)	
FAT1	chr4	187549706	NM_005245	c.G4535A	p.G1512D	missense	32.00	1.00	Disease_causing	1.00	0.00	1 (2.04%)	
HNF1A	chr12	121432085	NM_000545	c.C832T	p.R278W	missense	18.83	1.00	Disease_causing	1.00	0.00	1 (2.04%)	
MAP3K1	chr5	56178359	NM_005921	c.C3332T	p.T1111I	missense	21.10	1.00	Disease_causing	0.99	0.00	1 (2.04%)	
MAX	chr14	65569055	NM_001271068	c.G3A	p.M1I	missense	25.70	—	—	0.97	0.00	1 (2.04%)	
MSH6	chr2	48027683	NM_001281492	c.A2171T	p.K724M	missense	14.99	1.00	Disease_causing	1.00	0.00	1 (2.04%)	
MTOR	chr1	11188182	NM_004958	c.C5912T	p.A1971V	missense	33.00	1.00	Disease_causing	0.99	0.00	1 (2.04%)	
NOTCH2	chr1	120459205	NM_024408	c.G6140A	p.R2047Q	missense	26.50	1.00	Disease_causing	0.98	0.00	1 (2.04%)	
NOTCH2	chr1	120510804	NM_024408	c.G1160A	p.G387E	missense	28.90	1.00	Disease_causing	1.00	0.03	1 (2.04%)	
POLE	chr12	133249248	NM_006231	c.G1651A	p.V551I	missense	36.00	1.00	Disease_causing	0.99	0.00	1 (2.04%)	
SETD2	chr3	47058659	NM_014159	c.C7619A	p.T2540N	missense	23.60	1.00	Disease_causing	0.99	0.00	1 (2.04%)	
SETD2	chr3	47164922	NM_014159	c.C1204T	p.R402W	missense	15.70	0.99	Disease_causing	0.92	0.00	1 (2.04%)	
TSC1	chr9	135797294	NM_001162427	c.A422G	p.Y141C	missense	28.40	1.00	Disease_causing	1.00	0.00	1 (2.04%)	
TSC2	chr16	2131629	NM_001077183	c.C3512T	p.P1171L	missense	18.82	1.00	Disease_causing	1.00	0.01	1 (2.04%)	
MAX	chr14	65560500	NM_002382.4	c.C97T	p.R33^a^	stop-gain	36.00	1.00	Disease causing	-	-	1 (2.04%)	ANNOVAR
MSH6	chr2	48033607	NM_000179.2	c.A3818G	p.N1273S	missense	16.35	1.00	Disease_causing	0.99	0.19	1 (2.04%)	ANNOVAR
ARF1	chr1	228285611	NM_000249	c.T443C	p.L148P	missense	21.50	1.00	Disease_causing	1.00	0.00	1 (2.04%)	Metastatsis
PTGS1	chr9	125141063	NM_001271165	c.A35G	p.N12S	missense	13.39	1.00	Disease_causing	0.01	0.08	1 (2.04%)	Metastatsis
CHRNA5	chr15	78882925	NM_000745.3	c.G1192A	p.D398N	missense	10.45	0.00	polymorphism_automatic	0.01	0.18	3 (6.12%)	ANNOVAR
BRCA2	chr13	32893317	NM_000059.3	c.C171A	p.Y57^a^	stop-gain	28.90	1.00	Disease_causing_automatic	-	-	1 (2.04%)	ANNOVAR
CAGE1	chr6	7329448	NM_001170693	c.G2425C	p.D809H	missense	-	1.00	polymorphism	-	-	1 (2.04%)	Metastatsis
PTPRF	chr1	44058227	NM_002840	c.G1768A	p.V590M	missense	22.90	0.98	Disease_causing	0.51	0.17	1 (2.04%)	Metastatsis

a Metastatsis: variants identified in the metastatic tumors; ANNOVAR: SNVs (frequency <5%) with potential clinical significance in other diseases according to ANNOVAR software.

We collected two paired primary and metastatic tumors from two GIST patients, and observed several novel SNVs in the metastatic tumors but not in the primary tumors, including *ARF1* (c.T443C, p. R148C), *CAGE1* (c.G2425C, p.Q809H), *PTGS1* (c.A35G, p.N12S), and *PTPRF* (c.G1768A, p.V590M) (Table 3). We also analyzed SNVs (frequency <5%) with potential clinical significance (risk factor, pathogenic, or association) in diseases other than GIST using ClinVar tool of ANNOVAR software, and identified several variants, including *BRCA2* (rs201523522, p.Y57*), *CHRNA5* (rs16969968, p.D398N), *MAX* (rs387906651, p.R33*), and *MSH6* (rs201830316, p.N1273S) (Table 3 and Supplementary Table S6). In addition, to test the reliability of these results, we randomly chose some SNVs for Sanger sequencing validation, and confirmed these variants in GISTs (Figure 3B).

To further analyze whether these 34 genes have been identified in GIST, we summarized the somatic mutation profile in GISTs in COSMIC (https://cancer.sanger.ac.uk/cosmic). A total of 248 genes were mutated in the GIST cohort of COSMIC, including 19 genes identified in our GIST cohort (Table 3, Supplementary Table S7). However, most of these mutation sites were different in our cohort from those in the COSMIC GIST cohort, suggesting that these genes lack “hot site” mutations in GISTs.

### Pathway Enrichment Analyses of Mutant Genes in GIST

**FIGURE 4:**
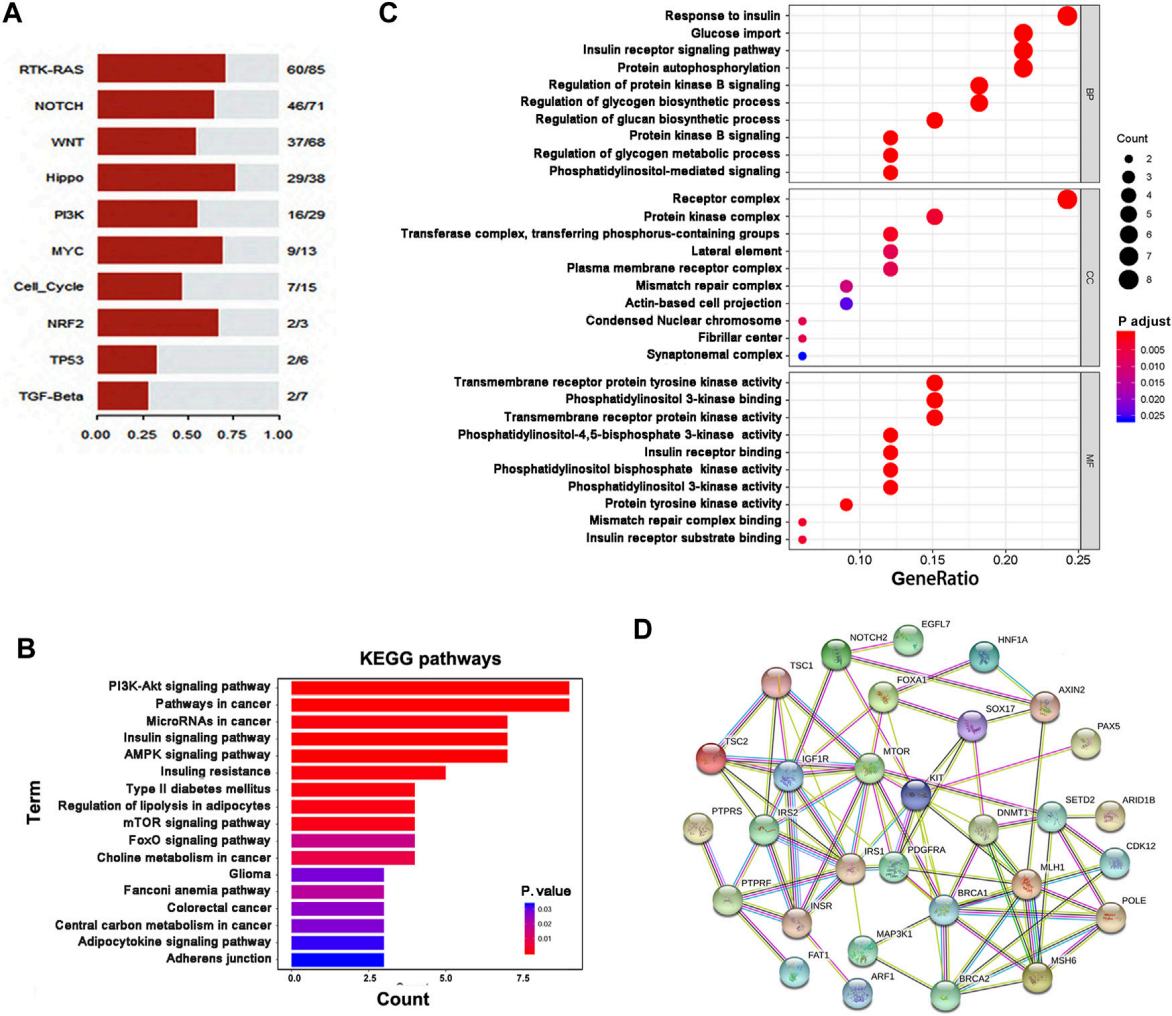
Pathway enrichment analyses of mutant genes in GIST. **(A)** Pathway enrichment of mutant genes in ten canonical oncogenic signaling pathways. **(B,C)** Enrichment analyses of gene function were performed based on 36 genes using the KEGG **(B)** or GO **(C)** pathway database. **(D)**Protein-protein interaction (PPI) networks were constructed based on 34 genes with mutations in GIST as well as KIT and PDGFRA using STRING software.

First, we enriched all mutant genes in ten canonical oncogenic signaling pathways (Sanchez-Vega et al., 2018) and observed obvious gene enrichment of these genes in these pathways, especially the RTK-RAS, Notch, Wnt, Hippo, and PI3K pathways (Figure 4A). We then analyzed 36 genes, including *KIT*, *PDGFRA* and 34 other genes with novel mutations or potential clinical significance (Table 3), using KEGG and GO pathway enrichment analyses, and showed that these genes were enriched in several important cancer- or metabolism-related signaling pathways, including PI3K-Akt, mTOR, AMPK, FoxO, and insulin signaling (Figures 4B, C). Of note, some key genes (*MLH1*, *MSH6*, *BRCA1*, *BRCA2,* and *POLE*) involved in DNA repair were frequently mutated in GIST, suggesting that deficiency in DNA repair may take part in the development and progression of GIST. In addition, some metabolism-related genes were also mutated in GIST, including *IGF1R*, *INSR*, *IRS1*, *IRS2*, *MAX*, *MTOR*, *TSC1,* and *TSC2*. To better understand the potential relationships among the proteins encoded by these 36 genes, these proteins were used to construct PPI networks, and a PPI network including 30 genes was obtained, suggesting that most of these proteins are functionally associated (Figure 4D).

### Mutation Validation in an Independent GIST Cohort

Based on the aforementioned results (Table 3 and Figure 4D), we selected 24 novel SNVs in 22 genes which were included in the pathway enrichment analyses, the PPI network and have not been reported to be associated with GIST. These variants were further validated in an expanded GIST cohort (Supplementary Table S4) using the SNaPshot SNP method. Of the 22 genes, *ARID1B* was abandoned due to the failure of PCR amplification. The validation results showed that six of 23 SNVs were further observed in the independent GIST cohort (Table 4). Of them, *MLH1*, *MSH6* and *BRCA1* are key DNA repair genes, whereas *ARF1* is a member of *RAS* superfamily. In addition, *IRS1* is associated with type II diabetes, susceptibility to insulin resistance and tumorigenesis (Choi et al., 2019). Interestingly, of the six variants, *BRCA1* c.T2425C appeared to be a polymorphism, whereas all of the other five resulted in protein function alteration (disease causing) based on the predictions by AlloDriver (http://mdl.shsmu.edu.cn/ALD/module/mainpage) and MutationTaster.

### Preliminary Analyses of CNV and TMB

**FIGURE 5:**
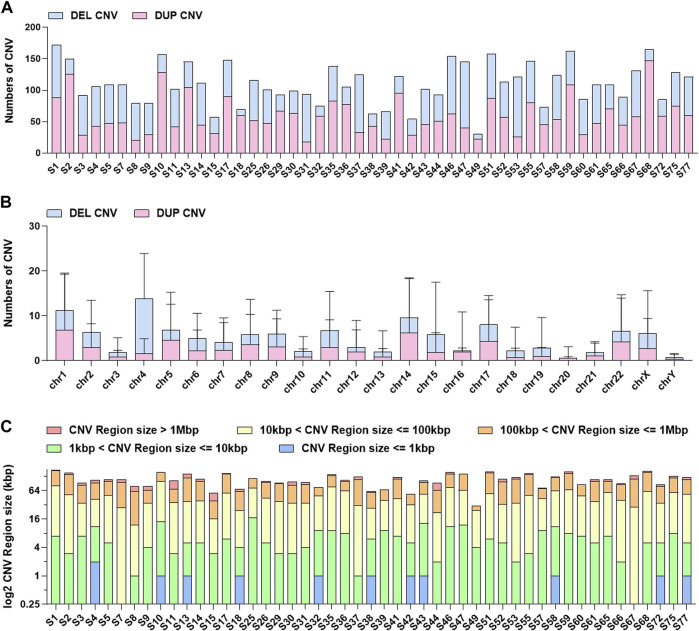
The distribution information of CNVs in 49 GISTs. **(A)**The number distribution of CNVs in each GIST. **(B)** The distribution of CNVs in each chromosome. **(C)** The length distribution of CNVs in each GIST.

We performed CNV analyses using the bioinformatics read-depth-based tool XHMM. Firstly, we evaluated the overall distribution of CNVs (Figure 5). In terms of the CNV distribution in the genome, the number of CNVs on chromosomes 1, 4, 14, and 17 was high and the CNVs on chromosome 4 were mainly deletion CNVs. It has been reported that chromosome 1 and chromosome 4 abnormality is associated with stomach cancer (Corless et al., 2014; Joensuu et al., 2017). Analyses of CNV length distribution showed that the number of CNVs between 1 and 100 KB accounted for the majority of all CNVs. Considering the accuracy of CNV testing software, the verification experiment needs to be further carried out, especially for the CNVs with a length larger than 1 MB. We further evaluated in detailed the CNV status in five important DNA damage response (DDR)-related pathways, including Mismatch Repair (MMR), base Excision Repair, Nucleotide Excision Repair, Homologous Recombination Repairand Nonho-mologous End-joining (Jie et al., 2018) (Supplementary Table S8). The results showed that the CNV-mediated abnormal DDR gene expression may result in defects in DNA damage repair mechanisms (Supplementary Table S9). It is noteworthy that more than two samples carried CNVs located in APTX, PARP1, RPA1, TOP3B, and POLR2L.

**TABLE 4:** Validation of SNVs using SNaPshot SNP assay in an independent GIST cohort (n = 97).

Genes	Mutation site	Amino acid change	Mutation ratio % (n/n)
ARF1	c.T443C	p.L148P	1.03% (1/97)
BRCA1	c.T2425C	p.Y809H	7.29% (7/96)
IRS1	c.C1360T	p.P454S	1.03% (1/97)
MLH1	c.C649T	p.R217C	1.10% (1/91)
MLH1	c.T428A	p.V143D	6.25% (6/96)
MSH6	c.A2171T	p.K724M	1.03% (1/97)

In our study, we chose somatic mutations to calculate TMB scores and somatic Signatures. Due to lack of stringent filtration of germline mutations, calculated TMBs seemed high with 15–19 mutations/MB in majority of tumors; no significant difference was observed between metastasis samples and primary tumors (Supplementary Figure S2). We also identified two known signature (Signature 6 and 15) (Supplementary Figure S3, S4),which had been observed in tumors with microsatellite instability and may be associated withDNA MMR deficiency (Alexandrov et al., 2015). It may provide some clues to clarify the relationship between DDR/MMR and GIST.

## Discussion

As the main drivers of GIST, gain-of-function mutations in *KIT* or *PDGFRA* have been identified in 85–95% of GIST tumors. At present, PCR-based mutation analyses are still extensively used in clinical practice, and provide sequence information in hot-spot sites or regions of DNA targets. However, high drug resistance and relapse ratios were observed in GIST patients, especially those with risk factors, suggesting that additional genes and pathways may be related to the development, progression and chemoresistance of GIST. The NGS-based techniques open up new opportunities by offering mutation analysis at the genome level with large sequencing depth and a low requirement of input DNA amount. In this study, we comprehensive analyzed genomic changes in GIST using WES, and identified a series of novel variants in *KIT/PDGFRA* or other tumor-related genes, and these genes are enriched in several DNA repair- or metabolism-related pathways. CNV analyses also suggest a potential relationship between DNA repair and GIST. These data may provide novel clues to understand the development and progression of GIST from an omics viewpoint.

Approximately 80% of GIST tumors harbor an identifiable gain-of-function *KIT* mutation, and more than half of driver mutations are located in exon 11 that encodes the juxtamembrane domain responsible for inhibiting receptor dimerization (activation) when the SCF ligand is absent (Shen et al., 2020). Approximately 20% of *KIT* mutations in GISTs are located in exon nine encoding the extracellular domain, which also results in ligand-independent receptor dimerization. In this study, a total of 30 different mutations were identified in exon 11 in 40 GIST tumors (81.62%), showing the extensive and diverse mutations of *KIT* exon 11. As expected, our results showed that most *KIT* exon 11 mutations were deletions (57.5%). In contrast, only two types of exon nine mutations located in the same site were observed in nine patients (19.15%). Interestingly, the most classical exon nine mutation (p.Ala502_Tyr503dup), which is mainly identified in GISTs in Caucasian patients, was not observed in our GIST cohort, suggesting that GISTs in Asian patients may harbor different mutational features of *KIT*.

CD117 staining is a standard assay for GIST diagnosis. According to IHC staining, the protein expression of CD117 was positive in 48 tumors except one with a KIT exon 11 deletion mutation (p.555_573del), and this patient relapsed after imatinib adjuvant therapy. Whether the deletion of 13 amino acids in exon 11 was associated with the chemoresistance to imatinib is unclear; however, others suggested that *KIT* exon 11 mutations involving codons 557/558 represent a novel GIST subgroup with increased malignant phenotypes and are associated with poor prognosis (Kontogianni-Katsarou et al., 2008). In addition, the negative expression of CD117 protein may relate to imatinib resistance, and the underling mechanism should be investigated in future studies. Although previous clinical trials have shown that GIST patients with *KIT* exon 11 mutations may obtain more benefits from adjuvant imatinib therapy (Corless et al., 2014; Joensuu et al., 2017), more detailed analyses should be considered to evaluate the functional and clinical roles of different mutation types of exon 11.

Mutant genes identified in this study were subject to pathway enrichment analyses using different methods. We revealed that, in addition to some canonical oncogenic signaling pathways, including RTK-RAS, Notch, Wnt, Hippo, PI3K-Akt, and mTOR, these genes alsoenriched in several important DNA repair- or metabolism-related signaling pathways. CNV analyses also suggest potential relationship between DDR/MMR and GIST. DNA repair-related signaling pathways play key roles in tumor susceptibility by maintaining genomic integrity, and their significance in GIST is largely unclear. Saito et al. reported that *MLH1* was hypermethylated in GIST (Saito et al., 2008). A recent paper reported a GIST case who harbored a *PDGFRA* (p.Trp559_Arg560del) and a *MLH1* (p.Met524Ile) mutation (Kobayashi et al., 2019). Ravegnini et al. investigated the influence of polymorphisms in several DNA repair genes on GIST susceptibility and characteristics, and showed that *XPD* rs13181, *hOGG1* rs1052133 and *XPF* rs1800067 were associated with GIST susceptibility, whereas *XPA* rs1800975 and rs2808668 were associated with tumor size, tumor metastasis and mitotic index (Ravegnini et al., 2016). Here, we revealed a series of novel variations in DNA repair genes. Some mutations in these genes have been reported to increase tumor susceptibility in certain human cancers, including hereditary nonpolyposis colon cancer or other hereditary cancer-predisposing syndromes. We showed that 12.77% of *KIT*-mutant GIST tumors harbored different *MLH1* mutations. In addition, we observed that one GIST patient with *MSH6* mutation was diagnosed with endometrial cancer.

More than 80% of *BRCA1/2* mutation carriers develop breast and/or ovarian cancer during their lifetime (Rebbeck et al., 2015). In addition to breast and ovarian cancers, *BRCA1/2* mutations increase risks for other cancer types, including pancreatic cancer, prostate cancer, and colorectal cancer (Varol et al., 2018). Moreover, breast cancer patients with *BRCA1/2* mutations show an obviously elevated risk of other or secondary malignancies. Parikh and others reported that alterations of DDR genes are relatively common in tubular gastrointestinal carcinomas (Parikh et al., 2019). Their data showed that *ARID1A* (9.2%), ATM (4.7%), *BRCA2* (2.3%), and *BRCA1* (1.1%) were the most commonly altered DDR genes in this cohort of 17486 cases. In this study, in addition to two novel SNVs, *BRCA1* p.Y809H (3/49) and *BRCA2* p.Y57*(1/49), a known pathologic SNV of *BRCA2* (c.3396delA, p.K1132Nfs) (Hereditary cancer-predisposing syndrome) was also identified in 44.91% (22/49) of GIST tumors (Supplementary Table S6). Approximately half of GISTs (51.06%) harbor at least one of these three potential disease-causing mutations, suggesting that *BRCA1/2* mutations are important risk factors for GIST. In addition, we also found five SNVs of *ATM* in five cases with uncertain significance or conflicting interpretations of pathogenicity (Supplementary Table S6). Emerging data suggest that an impaired DNA repair ability or certain DNA damage events indicate sensitivity to immune checkpoint blockade in cancers, and therapeutic implications of dDDR (DDR defect) and genomic instability are highlighted by recent clinical practices.

The high instability observed in mismatch repair deficiency is associated with a high TMB, a well-known predictive biomarker for immune checkpoint inhibitors. DDR or DNA repair-related genes are frequently observed in GISTs, suggesting that immune checkpoint blockade may have promising clinical application in these GIST subpopulations (Seifert et al., 2017).

Activation of the PI3K/AKT/mTOR pathway, a key downstream target pathway of KIT/PDGFRA, has been shown to be a crucial survival pathway in imatinib-resistant GISTs (Bauer et al., 2007). Interestingly, our data revealed many mutations in genes of this signaling pathway, including *IGF1R*, *MTOR*, *TSC1*, *FLT4*, *TSC2*, *IRS1*, *INSR*, *and BRCA1*, suggesting that these KIT downstream signaling intermediates may mediate resistance to imatinib or other KIT inhibitors. Of them, *IGF1R* and *MTOR* have been reported to be associated with the development and progression of GIST (Li et al., 2013). In addition to its association with type II diabetes and insulin resistance, *IRS1* also promotes tumorigenesis by regulating the ErbB-PI3K-AKT signaling cascade (Choi et al., 2019). Some variations in *IRS1* have been reported to be related to increased cancer risk (Slattery et al., 2004; Maglio et al., 2013). Several clinical trials targeting PI3K/AKT/mTOR signaling are currently being investigated as promising targeted therapy strategies for GIST (Duan et al., 2020). The AMPK pathway, a key regulator of cellular energy metabolism, is closely correlated with several key cellular survival signaling pathways such as mTOR and PI3K/AKT. At the same time, AMPK is tightly involved in cancer chemoresistance by regulating autophagy and cancer stemness (Zadra et al., 2015; Wang et al., 2016). In this study, mutations identified in genes (i.e., *TSC1*, *TSC2*, *MTOR*, and *IRS1*) that were shared by the three pathways (PI3K/AKT, mTOR and AMPK), strongly suggested that targeting these pathways or genes may present promising strategies for GIST prevention and treatment. Metabolism reprograming is an important feature of cancer cells. Frequent mutations in metabolism-related genes, especially insulin- and diabetes-related pathways in this study, suggest a potential pathologic role of these genes in GIST and a possible association between GIST and diabetes mellitus.

Interestingly, a missense mutation of *ARF1* (p.L148P), identified in a metastatic GIST tumor, was further verified in a relapsed GIST tumor of the test cohort, suggesting its potential role in GIST progression and therapeutic resistance. Several papers have reported that *ARF1* could promote tumor development and metastasis in other cancer types, including breast cancer (Haines et al., 2014), cervical cancer (Xu and Zhang, 2020), prostate cancer (Davis et al., 2016), head and neck squamous cell carcinoma (Vo-Hoang et al., 2020). The pathologic role of this mutation is unclear and deserves for further investigations.

Protein tyrosine phosphatase (PTP) plays important roles in tumorigenesis and progression by regulating cell proliferation, apoptosis, migration, and invasion. Interestingly, we identified some novel mutations in several members of PTP family, including *PTPRS* (p.Y296N) and *PTPRF* (p.V590M). In addition, a known pathogenic mutation of *PTPRJ* (p.Gln276Pro) (colorectal cancer) was also observed in 40.1% (20/49) GIST tumors (Supplementary Table S6). Many PTPs are important regulators of the RAS/ERK pathway and play tumor suppressive roles. For example, PTPRE, PTPRJ and PTPRS could inhibit ERK activation (Toledano-Katchalski et al., 2003; Davis et al., 2018). Approximately 10% of colorectal cancers harbor native mutations in *PTPRS*, and inactivation of *PTPRS* promotes ERK and AKT activation, resulting in enhanced RAS and EGFR activity in colorectal cancer (Davis et al., 2018). *PTPRS* loss promotes EGFR/PI3K pathway activation and modulates resistance to EGFR inhibition (Du and Grandis, 2015). PRTPRJ exerts tumor-inhibitory effects by negatively regulating mitogenic signals originating from several oncogenic receptor tyrosine kinases, including PDGFRA. Aberrant promoter hypermethylation or DNA copy number alterations have also been observed in several PTPs, including *PTPRM*, *PTPRT*, *PTPRR* and *PTPRZ1* (Laczmanska et al., 2013; Laczmanska et al., 2014). Several PTPs have been suggested as promising therapeutic targets for human diseases, especially cancers. These data suggest key roles of PTPs in human cancers, including GIST.

Identified mutant genes in this study also enriched in Notch, Wnt, and Hippo pathways. These pathways play important role in cancer development and progression by regulating cell proliferation, tumor metastasis and cancer stemness.

There are several limitations in this study. First, as a retrospective study, matched blood samples were not included, which made it difficult to differentiate germline mutations from somatic cell mutations and also resulted in high TMB results due to lack of stringent filtration of germline mutations. Second, the validation positive rate seems low, which may be due to discounted sensitivity of the SNaPshot SNP assay compared with the WES method. In addition, the low frequencies of these SNVs may also explain the relatively low reappearance of these SNVs in the validation cohort. Third, although mutations identified in this study were predicted to be disease-causing variants, their exact functional significance and pathogenicity in GIST was not evaluated.

In conclusion, by systemic mutation analyses of *KIT*-mutant Chinese GISTs, we revealed some novel *KIT* mutations. We also identified some novel mutations in genes that are closely related to DNA repair-, cancer- or metabolism-related signaling pathways, including PI3K/Akt, mTOR, AMPK, FoxO, and insulin signaling. These data widen the spectrum of known gene mutations in GIST and suggest novel strategies from the angles of metabolism and DNA repair for GIST prevention and treatment.

## Data Availability

The datasets presented in this study can be found in online repositories. The names of the repository/repositories and accession number(s) can be found below: ScienceDB [DOI: 10.11922/sciencedb.01155].
