# Supplementary figures and images for: Identification of New Tumor-Related Gene Mutations in Chinese Gastrointestinal Stromal Tumors
Source: Front Cell Dev Biol. 2021 Nov 3;9:764275. doi: 10.3389/fcell.2021.764275 (PMC8595335; doi:10.3389/fcell.2021.764275)

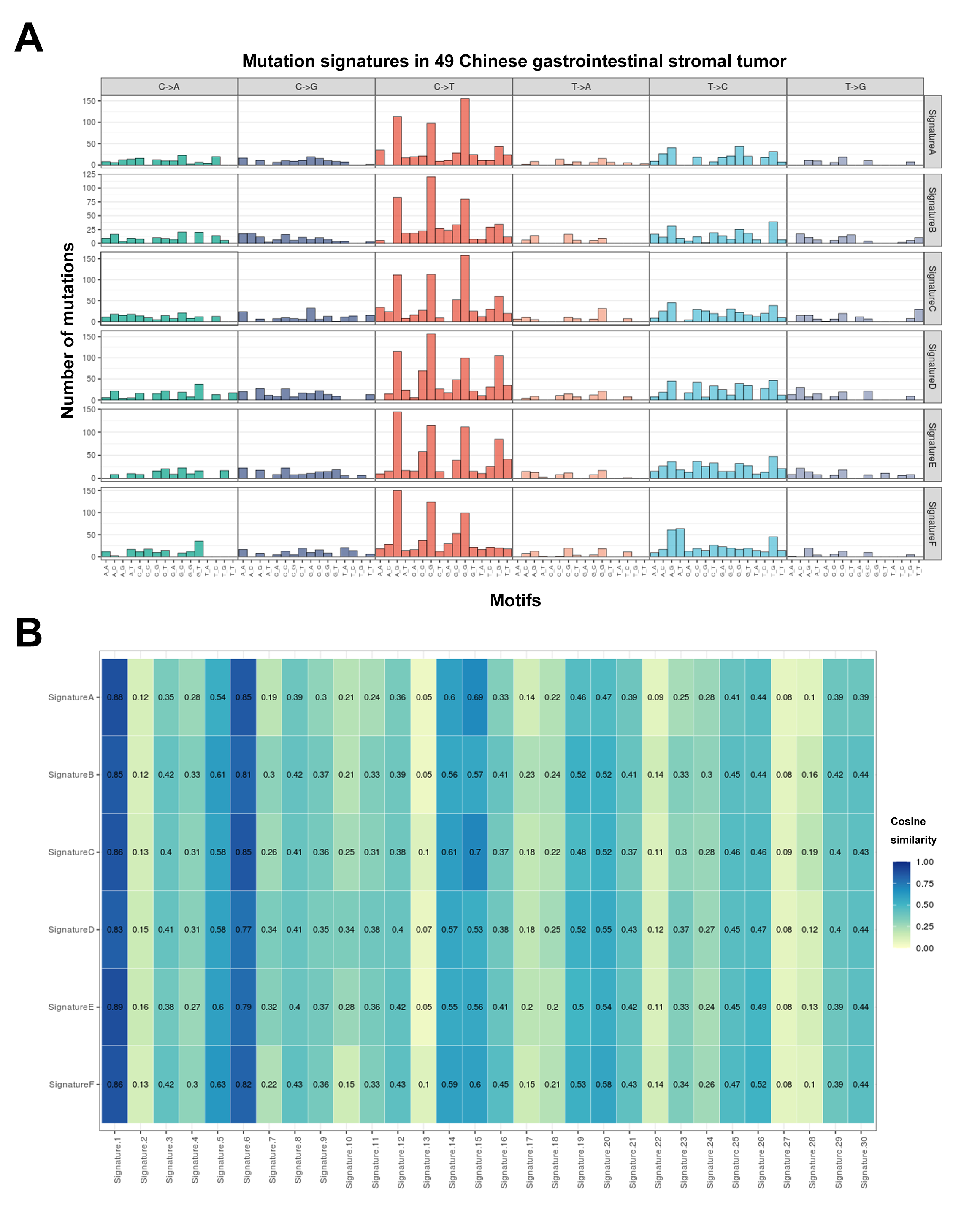

Supplement: Supplementary file 2 [file Image3.TIF]

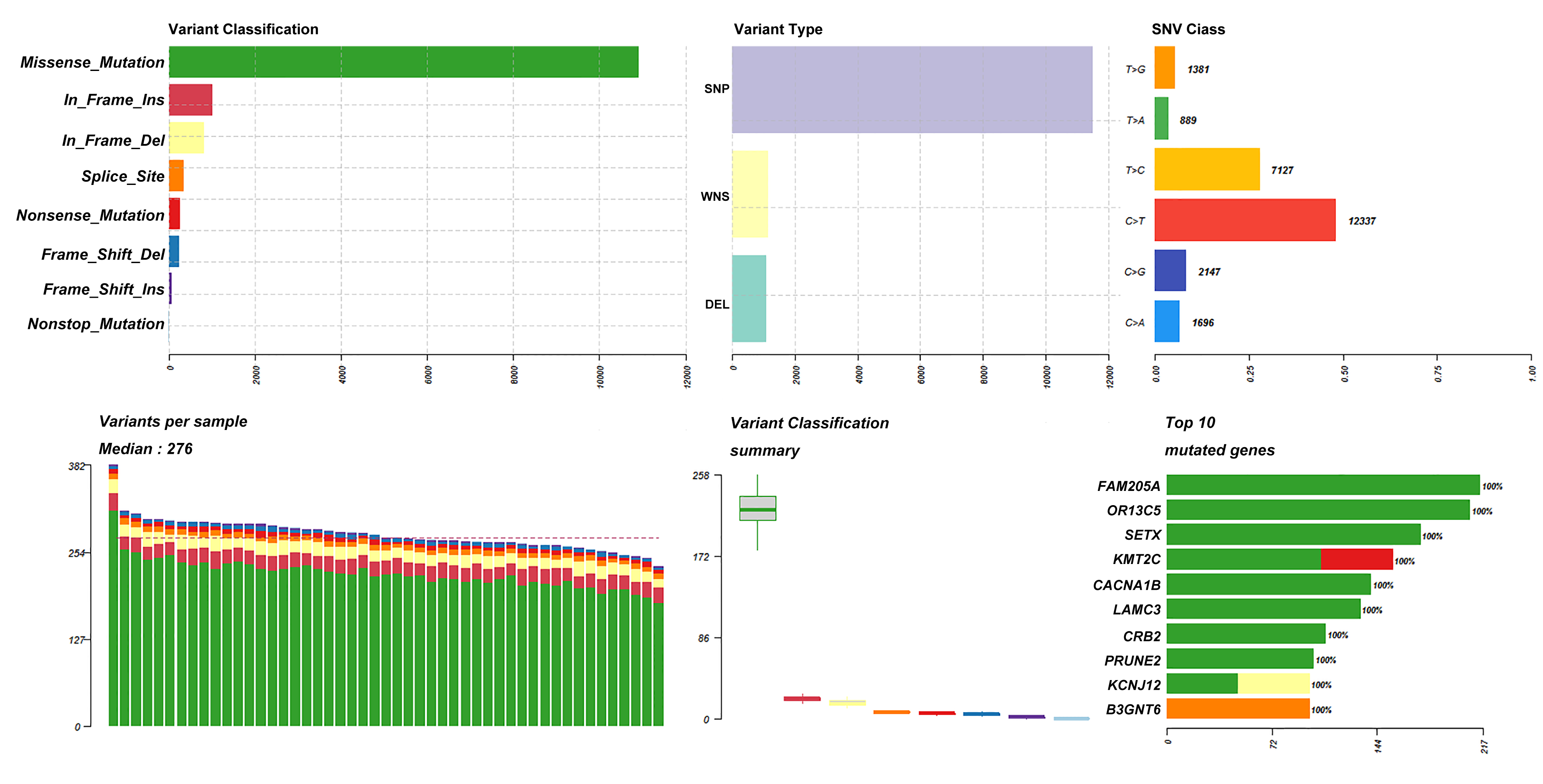

Supplement: Supplementary file 3 [file Image4.TIF]

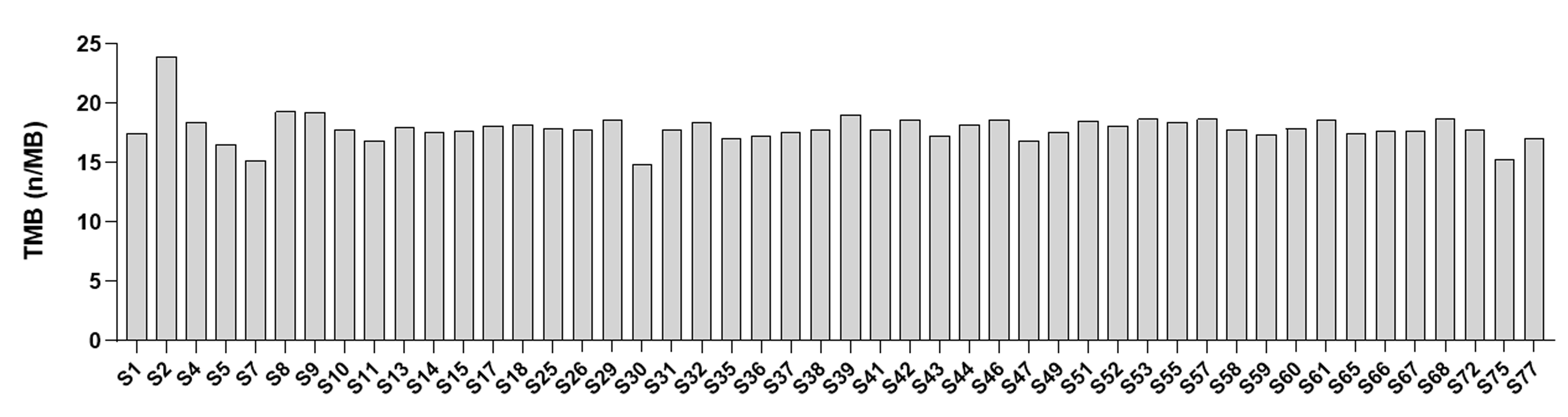

Supplement: Supplementary file 4 [file Image2.TIF]

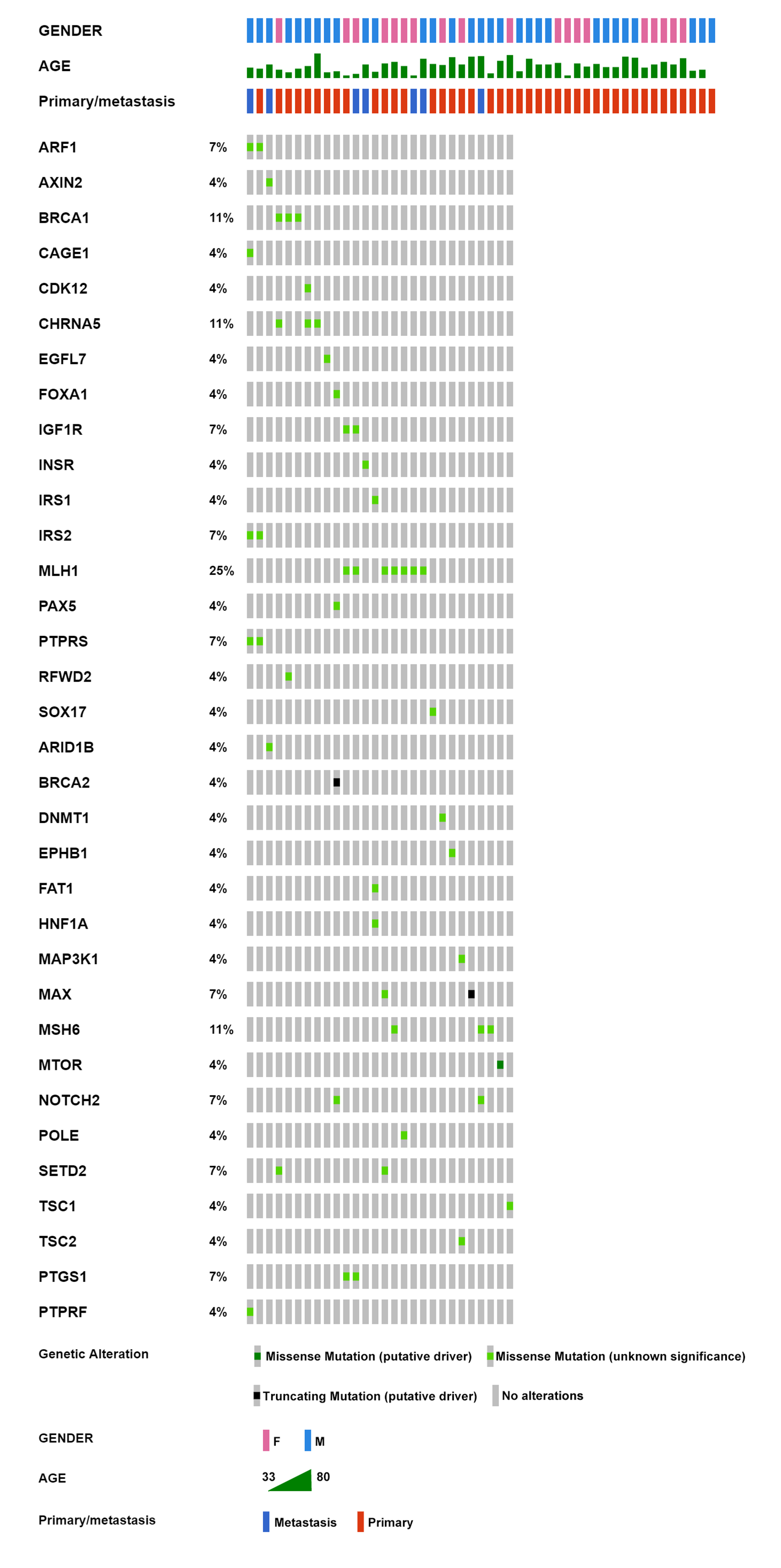

Supplement: Supplementary file 5 [file Image1.TIF]
