# Supplementary material for: Identification of New Tumor-Related Gene Mutations in Chinese Gastrointestinal Stromal Tumors
Source: Front Cell Dev Biol. 2021 Nov 3;9:764275. doi: 10.3389/fcell.2021.764275 (PMC8595335; doi:10.3389/fcell.2021.764275)
Supplement: Supplementary file 7 [file Table2.DOCX]

Supplementary Table 2. List of Genes Covered in MSK-IMPACT Panel.

| **Gene**  **Canonical Refseq transcript** | | | | | |
| --- | --- | --- | --- | --- | --- |
| *ABL1*  NM_005157 | *AKT1* NM_001014431 | *AKT2*  NM_001626 | *AKT3*  NM_005465 | *ALK*  NM_004304 | *ALOX12B* NM_001139 |
| *APC*  NM_000038 | *AR*  NM_000044 | *ARAF*  NM_001654 | *ARID1A* NM_006015 | *ARID1B* NM_020732 | *ARID2*  NM_152641 |
| *ARID5B* NM_032199 | *ASXL1*  NM_015338 | *ASXL2*  NM_018263 | *ATM*  NM_000051 | *ATR*  NM_001184 | *ATRX*  NM_000489 |
| *AURKA* NM_003600 | *AURKB* NM_004217 | *AXIN1*  NM_003502 | *AXIN2*  NM_004655 | *AXL*  NM_021913 | *B2M*  NM_004048 |
| *BAP1*  NM_004656 | *BARD1* NM_000465 | *BBC3* NM_001127240 | *BCL2*  NM_000633 | *BCL2L1* NM_138578 | *BCL2L11* NM_138621 |
| *BCL6*  NM_001706 | *BCOR* NM_001123385 | *BLM*  NM_000057 | *BMPR1A* NM_004329 | *BRAF*  NM_004333 | *BRCA1* NM_007300 |
| *BRCA2* NM_000059 | *BRD4*  NM_058243 | *BRIP1*  NM_032043 | *BTK*  NM_000061 | *CARD11* NM_032415 | *CASP8* NM_001080125 |
| *CBFB* NM_022845 | *CBL* NM_005188 | *CCND1* NM_053056 | *CCND2* NM_001759 | *CCND3* NM_001760 | *CCNE1* NM_001238 |
| *CD274* NM_014143 | *CD276* NM_001024736 | *CD79B* NM_001039933 | *CDC73* NM_024529 | *CDH1*  NM_004360 | *CDK12* NM_016507 |
| *CDK4* NM_000075 | *CDK6* NM_001145306 | *CDK8* NM_001260 | *CDKN1A* NM_078467 | *CDKN1B* NM_004064 | *CDKN2A p16 INK4A:* NM_000077  *P14ARF:* NM_058195 |
| *CDKN2B* NM_004936 | *CDKN2C* NM_078626 | *CHEK1* NM_001274 | *CHEK2* NM_007194 | *CIC*  NM_015125 | *CREBBP* NM_004380 |
| *CRKL*  NM_005207 | *CRLF2*  NM_022148 | *CSF1R*  NM_005211 | *CTCF*  NM_006565 | *CTLA4*  NM_005214 | *CTNNB1* NM_001904 |
| *CUL3*  NM_003590 | *DAXX* NM_001141970 | *DCUN1D1* NM_020640 | *DDR2*  NM_006182 | *DICER1* NM_030621 | *DIS3*  NM_014953 |
| *DNMT1* NM_001379 | *DNMT3A* NM_022552 | *DNMT3B* NM_006892 | *DOT1L* NM_032482 | *E2F3*  NM_001949 | *EED*  NM_003797 |
| *EGFL7*  NM_201446 | *EGFR*  NM_005228 | *EIF1AX* NM_001412 | *EP300*  NM_001429 | *EPCAM* NM_002354 | *EPHA3* NM_005233 |
| *EPHA5* NM_004439 | *EPHB1* NM_004441 | *ERBB2*  NM_004448 | *ERBB3*  NM_001982 | *ERBB4*  NM_005235 | *ERCC2*  NM_000400 |
| *ERCC3*  NM_000122 | *ERCC4*  NM_005236 | *ERCC5*  NM_000123 | *ERG*  NM_182918 | *ESR1* NM_001122740 | *ETV1* NM_001163147 |
| *ETV6*  NM_001987 | *EZH2*  NM_004456 | *FAM123B* NM_152424 | *FAM175A* NM_139076 | *FAM46C* NM_017709 | *FANCA* NM_000135 |
| *FANCC* NM_000136 | *FAT1*  NM_005245 | *FBXW7* NM_033632 | *FGF19*  NM_005117 | *FGF3*  NM_005247 | *FGF4*  NM_002007 |
| *FGFR1* NM_001174067 | *FGFR2*  NM_000141 | *FGFR3*  NM_000142 | *FGFR4*  NM_213647 | *FH*  NM_000143 | *FLCN*  NM_144997 |
| *FLT1*  NM_002019 | *FLT3*  NM_004119 | *FLT4*  NM_182925 | *FOXA1* NM_004496 | *FOXL2*  NM_023067 | *FOXP1* NM_001244814 |
| *FUBP1* NM_003902 | *GATA1* NM_002049 | *GATA2* NM_032638 | *GATA3* NM_002051 | *GNA11* NM_002067 | *GNAQ*  NM_002072 |
| *GNAS*  NM_000516 | *GREM1* NM_013372 | *GRIN2A* NM_001134407 | *GSK3B* NM_002093 | *H3F3C* NM_001013699 | *HGF*  NM_000601 |
| *HIST1H1C* NM_005319 | *HIST1H2BD* NM_021063 | *HIST1H3B* NM_003537 | *HNF1A* NM_000545 | *HRAS* NM_001130442 | *ICOSLG* NM_015259 |
| *IDH1*  NM_005896 | *IDH2*  NM_002168 | *IFNGR1* NM_000416 | *IGF1* NM_001111285 | *IGF1R*  NM_000875 | *IGF2* NM_001127598 |
| *IKBKE*  NM_014002 | *IKZF1*  NM_006060 | *IL10*  NM_000572 | *IL7R*  NM_002185 | *INPP4A* NM_001134224 | *INPP4B* NM_001101669 |
| *INSR*  NM_000208 | *IRF4*  NM_002460 | *IRS1*  NM_005544 | *IRS2*  NM_003749 | *JAK1*  NM_002227 | *JAK2*  NM_004972 |
| *JAK3*  NM_000215 | *JUN*  NM_002228 | *KDM5A* NM_001042603 | *KDM5C* NM_004187 | *KDM6A* NM_021140 | *KDR*  NM_002253 |
| *KEAP1*  NM_203500 | *KIT*  NM_000222 | *KLF4*  NM_004235 | *KRAS*  NM_033360 | *LATS1*  NM_004690 | *LATS2*  NM_014572 |
| *LMO1*  NM_002315 | *MAP2K1* NM_002755 | *MAP2K2* NM_030662 | *MAP2K4* NM_003010 | *MAP3K1* NM_005921 | *MAP3K13* NM_004721 |
| *MAPK1* NM_002745 | *MAX*  NM_002382 | *MCL1*  NM_021960 | *MDC1*  NM_014641 | *MDM2* NM_002392 | *MDM4* NM_002393 |
| *MED12* NM_005120 | *MEF2B* NM_001145785 | *MEN1*  NM_000244 | *MET*  NM_000245 | *MITF*  NM_198159 | *MLH1*  NM_000249 |
| *MLL* NM_001197104 | *MLL2*  NM_003482 | *MLL3*  NM_170606 | *MPL*  NM_005373 | *MRE11A* NM_005591 | *MSH2*  NM_000251 |
| *MSH6*  NM_000179 | *MTOR*  NM_004958 | *MUTYH* NM_001048172 | *MYC*  NM_002467 | *MYCL1* NM_001033082 | *MYCN*  NM_005378 |
| *MYD88* NM_002468 | *MYOD1* NM_002478 | *NBN*  NM_002485 | *NCOR1* NM_006311 | *NF1* NM_001042492 | *NF2*  NM_000268 |
| *NFE2L2* NM_006164 | *NKX2-1* NM_001079668 | *NKX3-1* NM_006167 | *NOTCH1* NM_017617 | *NOTCH2* NM_024408 | *NOTCH3* NM_000435 |
| *NOTCH4* NM_004557 | *NPM1*  NM_002520 | *NRAS*  NM_002524 | *NSD1*  NM_022455 | *NTRK1* NM_002529 | *NTRK2* NM_006180 |
| *NTRK3* NM_001012338 | *PAK1*  NM_002576 | *PAK7*  NM_177990 | *PALB2*  NM_024675 | *PARK2* NM_004562 | *PARP1* NM_001618 |
| *PAX5*  NM_016734 | *PBRM1*  NM_018313 | *PDCD1* NM_005018 | *PDGFRA* NM_006206 | *PDGFRB* NM_002609 | *PDPK1* NM_002613 |
| *PHOX2B* NM_003924 | *PIK3C2G* NM_004570 | *PIK3C3* NM_002647 | *PIK3CA* NM_006218 | *PIK3CB* NM_006219 | *PIK3CD* NM_005026 |
| *PIK3CG* NM_002649 | *PIK3R1* NM_181523 | *PIK3R2* NM_005027 | *PIK3R3* NM_003629 | *PIM1*  NM_002648 | *PLK2*  NM_006622 |
| *PMAIP1* NM_021127 | *PMS1*  NM_000534 | *PMS2*  NM_000535 | *PNRC1* NM_006813 | *POLE*  NM_006231 | *PPP2R1A* NM_014225 |
| *PRDM1* NM_001198 | *PRKAR1A* NM_212471 | *PTCH1* NM_000264 | *PTEN*  NM_000314 | *PTPN11* NM_002834 | *PTPRD* NM_002839 |
| *PTPRS*  NM_002850 | *RAC1* N  M_018890 | *RAD50* NM_005732 | *RAD51* NM_002875 | *RAD51B* NM_133509 | *RAD51C* NM_058216 |
| *RAD51D* NM_133629 | *RAD52* NM_134424 | *RAD54L* NM_001142548 | *RAF1*  NM_002880 | *RARA*  NM_000964 | *RASA1* NM_002890 |
| *RB1*  NM_000321 | *RBM10* NM_001204468 | *RECQL4* NM_004260 | *REL*  NM_002908 | *RET*  NM_020975 | *RFWD2* NM_022457 |
| *RHOA*  NM_001664 | *RICTOR* NM_152756 | *RIT1*  NM_006912 | *RNF43* NM_017763 | *ROS1* N  M_002944 | *RPS6KA4* NM_003942 |
| *RPS6KB2* NM_003952 | *RPTOR* NM_020761 | *RUNX1* NM_001754 | *RYBP*  NM_012234 | *SDHA*  NM_004168 | *SDHAF2* NM_017841 |
| *SDHB*  NM_003000 | *SDHC*  NM_003001 | *SDHD*  NM_003002 | *SETD2*  NM_014159 | *SF3B1*  NM_012433 | *SH2D1A* NM_002351 |
| *SHQ1*  NM_018130 | *SMAD2* NM_001003652 | *SMAD3* NM_005902 | *SMAD4* NM_005359 | *SMARCA4* NM_003072 | *SMARCB1* NM_003073 |
| *SMARCD1* NM_003076 | *SMO*  NM_005631 | *SOCS1*  NM_003745 | *SOX17*  NM_022454 | *SOX2*  NM_003106 | *SOX9*  NM_000346 |
| *SPEN*  NM_015001 | *SPOP* NM_001007228 | *SRC*  NM_198291 | *STAG2* NM_001042749 | *STK11*  NM_000455 | *STK40*  NM_032017 |
| *SUFU*  NM_016169 | *SUZ12*  NM_015355 | *SYK*  NM_003177 | *TBX3*  NM_016569 | *TERT*  NM_198253 | *TET1*  NM_030625 |
| *TET2* NM_001127208 | *TGFBR1* NM_004612 | *TGFBR2* NM_001024847 | *TMEM127* NM_001193304 | *TMPRSS2* NM_001135099 | *TNFAIP3* NM_006290 |
| *TNFRSF14* NM_003820 | *TOP1*  NM_003286 | *TP53*  NM_000546 | *TP63*  NM_003722 | *TRAF7*  NM_032271 | *TSC1*  NM_000368 |
| *TSC2*  NM_000548 | *TSHR*  NM_000369 | *U2AF1* NM_006758 | *VHL*  NM_000551 | *VTCN1* NM_024626 | *WT1*  NM_024426 |
| *XIAP*  NM_001167 | *XPO1*  NM_003400 | *YAP1* NM_001130145 | *YES1*  NM_005433 |  |  |
